# Palliative Care and Chronic Obstructive Pulmonary Disease (COPD) Readmissions: A Narrative Review

**DOI:** 10.7759/cureus.16987

**Published:** 2021-08-07

**Authors:** Wasey Ali Yadullahi Mir, Abdul Hasan Siddiqui, Vishesh Paul, Saad Habib, Shravani Reddy, Suman Gaire, Dhan B Shrestha

**Affiliations:** 1 Department of Internal Medicine, Mount Sinai Medical Center, Chicago, USA; 2 Pulmonary and Critical Care Medicine, University of Illinois at Urbana-Champaign, Champaign, USA; 3 Pulmonary and Critical Care Medicine, Carle Foundation Hospital, Urbana, USA; 4 Internal Medicine, Staten Island University Hospital/Northwell Health, Staten Island, USA; 5 Department of Internal Medicine, Rush University Medical Center, Chicago, USA; 6 Department of Emergency Medicine, Palpa Hospital, Palpa, NPL; 7 Department of Medicine, Mount Sinai Hospital, Chicago, USA

**Keywords:** quality of life, patient readmission, palliative care, terminal care, chronic obstructive pulmonary disease

## Abstract

Despite all the advances in the treatment and management of chronic obstructive pulmonary disease (COPD), COPD readmissions remain a major challenge nationwide. Increasing evidence suggests that palliative care involvement with a holistic approach towards end-of-life care can significantly improve outcomes related to the quality of life and survival for late-stage cancers and chronic progressive illnesses like COPD, chronic heart failure, and end-stage renal disease. Some studies have attempted to evaluate an association between the involvement of palliative care and readmission reduction, the effect of which remains elusive, especially with regards to COPD readmissions. This review examined the existing literature to analyze the relationship between palliative care involvement for COPD patients and its effect on COPD readmissions.

## Introduction and background

According to the World Health Organization (WHO), chronic obstructive pulmonary disease (COPD) is the third leading cause of death worldwide, affecting about 65 million people [1]. Patients admitted to the hospital with a COPD exacerbation have a median survival of two years, and 50% of them will be readmitted within six months. 10-20% of COPD patients are readmitted within the 30 days of discharge from the hospital, which roughly accounts for about $325 million per year in healthcare expenditures [2]. As a result, COPD was made a part of Medicare's Hospital Readmissions Reduction Program (HRRP) to address this problem. In 2014 Centers for Medicare and Medicaid Services started imposing financial penalties to hospitals with higher than expected all-cause 30-day rehospitalization rates after acute exacerbation of COPD (AECOPD) discharge [3,4]. Increasing evidence suggests that palliative care involvement with a holistic approach towards end-of-life care can significantly improve outcomes related to the quality of life and survival for late-stage cancers and chronic progressive illnesses like COPD, congestive heart failure (CHF), and end-stage renal disease (ESRD) [5,6]. However, the effect of palliative care in reducing COPD readmissions has not been well studied. In the light of existing literature, we aim to explore the various facets of palliative care and its potential influence on COPD readmission.

## Review

Palliative care and COPD

WHO defines palliative care as "the active total care of patients whose disease is not responsive to curative treatment". It further states that it should include an approach that improves patients' quality of life and supports the physical, psychosocial, and spiritual beliefs of families [7,8]. Multiple studies have shown that the symptom burden among COPD patients is much more than that seen in lung cancer or any other cancer. Therefore, there is a similar need for palliative care services for patients suffering from COPD [8]. Over the decades, there has been a gradual increase in the involvement of palliative care for COPD. The use of opiates to relieve dyspnea, oxygen therapy, pharmacotherapy for anxiety and depression, and cognitive behavioral therapy has been shown to improve patients' quality of life. It also gives a sense of satisfaction to the family by reducing the stress and pain of seeing their loved ones in agony [9,10].

Even though existing literature has emphasized that patient with COPD needs palliative care, the exact point at which this should be initiated is uncertain [11]. Also, there are no defined outcome measures that serve as targets for palliative care for these patients. The commonly tested outcome measures include but are not limited to symptom control, quality of life, the institution of advance directives, physical, social and psychological wellbeing. Some studies have also looked at objective measures like health care utilization near the end of life, readmission, and survival [8,12]. Unfortunately, none of these have been validated among COPD patients.

Duenk et al. conducted a pragmatic cluster-controlled trial in the Netherlands to assess the outcomes related to specialized palliative care for COPD patients admitted to the hospitals. Hospitals were selected for the intervention group based on a specialized palliative care team (SPCT), and hospitals were compared on baseline characteristics. Patients with COPD with poor prognoses were recruited during hospitalization for acute exacerbation. All patients received usual care while patients in the intervention group received additional proactive palliative care in monthly meetings with an SPCT. 228 patients (90 intervention and 138 control) were recruited, and at three months, 163 patients (67 intervention and 96 control) completed the St. George Respiratory Questionnaire (SGRQ). There was no significant difference in change scores of the SGRQ total at three months between groups (-0.79 [95% CI, -4.61 to 3.34], p=0.70). However, patients who received proactive palliative care experienced less impact of their COPD (SGRQ impact subscale) at six months (-6.22 [-11.73 to -0.71], p=0.04) and had more often made advance care planning (ACP) choices (adjusted odds ratio 3.26 [1.49-7.14], p=0.003). Other secondary outcomes were also not significantly different, and they concluded that proactive palliative care did not improve the overall quality of life of patients with COPD. However, patients more often made ACP choices, leading to a better quality of care toward the end of life [10].

Whenever and wherever the COPD outcomes are assessed for palliative care, the targets are more in terms of end-of-life care. In COPD, the complexity of the overall intervention is dictated by the disease severity, comorbidities, advanced age, and decline in cognitive status. The evaluation of palliative care needs in disease is conventionally comprised of four domains: physical, psychological, social, and spiritual. Physical outcomes may not be a target of palliative care as they do not usually improve with palliative care alone and usually require active medical interventions. Furthermore, when the functional status was evaluated in a subset of the study by Duenk et al., at discharge, it was demonstrated that those unable to walk at hospital discharge had a twofold risk of being readmitted within the next 30 days compared to those able to walk [12]. Physical outcome measures include general measures and disease-specific outcomes such as related to morbidity or disease-specific symptoms. Irrespective of the disease, commonly used psychological outcome measures are represented by anxiety/depression and cognitive deficit [13]. Social outcome measures are centered on caregiver burden (strain) and social status impairment. Spiritual outcome measures should include dignity, hope, religious insertion, and meaning in life. A list of possible outcome measures as a target for palliative care for COPD patients is presented in Table 1.

**Table 1:** Meaningful outcome measures in COPD palliative care

Categories of outcome measures	Variables
Physical outcome measures	a. Dyspnea b. Fatigue c. Other non-respiratory symptoms (mainly pain) d. Functional status e. Health status f. Frailty g. Disease-related morbidity: exacerbations
Psychological outcomes	a. Anxiety b. Depression c. Delirium and other cognitive deficits
Social outcome measures	a. Social support b. Caregiver burden
Spiritual outcome measures	a. Hope b. Meaning in life c. Dignity d. Religious belief

The frequency of exacerbation and admissions related to them has been used to indicate palliative care involvement for COPD. Still, the effect of palliative care to reduce COPD readmissions has not been tested adequately. Moreover, the studies of palliative care for COPD have not shown any reduction in COPD-related hospital readmissions. However, readmission reductions and health care utilization are more recently used as outcome measures for palliative care involvement in general, especially concerning prolonged medical illnesses like COPD, CHF, and ESRD [14,15].

Dignity was also evaluated in COPD patients with advanced disease. From a sample of 195 patients, about 13% had a significant loss of dignity irrespective of the presence of the end-of-life stage. This was related to psychological distress such as depression and anxiety and not the severity of the underlying disease itself. Furthermore, spiritual interventions tailored to improve dignity were demonstrated to improve spiritual wellbeing across various end-stage pathologies [15].

A systematic review conducted by Almagro et al. in 2017 showed that none of the suggested criteria to involve palliative care for COPD patients offer short- or long-term prognosis with sufficient reliability [16]. The primary reason for this is the unpredictability of the progression of COPD and the impact of comorbidities on the overall prognosis of the disease.

In summary, palliative care services may benefit patients with COPD in general, but there is a lack of data to support this hypothesis. A major obstacle could be the inconsistency amongst the outcome measures that are being tested. Nevertheless, the impact of palliative care involvement to prevent COPD readmissions can be a meaningful outcome concerning healthcare utilization, especially at the end of life [17]. 

The burden of COPD readmissions

One of the most critical risk factors associated with the decline of respiratory function is the AECOPD. This flare-up is characterized by acute worsening of respiratory symptoms requiring escalation of treatment. Clinically, a hospitalization for AECOPD is defined as a change in dyspnea, cough, or sputum quality. The most frequent cause of these exacerbations is respiratory viral and bacterial infections. AECOPD has been shown to increase the possibility of subsequent COPD-related hospitalizations and mortality and accounts for the majority of costs associated with COPD [18,19].

On average, one in five patients requires rehospitalization within 30 days of discharge after admission for AECOPD, imposing a substantial economic burden to the nation. Factors that have been associated with early readmission include premature discharge, inability to identify and manage comorbidities associated with COPD, poor discharge medication reconciliation, lack of family education on disease management, and lack of communication with outpatient physicians who will be handling patient care. In addition, the readmitted patients tend to have a longer length of stay in the hospital with a higher incidence of ICU use than those not readmitted [20].

About 10-20% of COPD patients are readmitted within a month of discharge, causing about $325 million per year in healthcare expenditures [2]. COPD was made a part of Medicare's Hospital Readmissions Reduction Program (HRRP) to address this problem. In 2014 Centers for Medicare and Medicaid Services started imposing financial penalties to hospitals with higher than expected all-cause 30-day hospital rehospitalization rates after AECOPD discharge. Hospitals then started implementing value-based health care with a target to reduce early readmissions. Considerable research has been carried out to estimate the risk of readmissions for COPD-related hospitalizations to reduce it. Approximately 10% to 55% of readmissions after an "index admission" for AECOPD may be preventable [2]. Inhaler education, an action plan in case of clinical deterioration, home visits to provide education, and assessing clinical status, post-acute rehabilitation including noninvasive ventilation and long-term oxygen therapy have been studied. However, none of these interventions have led to a promising result. We still lack an evidence-based approach in reducing hospital readmissions following AECOPD [2].

Underutilization of palliative care among COPD patients

Even though a few studies have shown the beneficial aspects of palliative care among COPD patients, there is substantial evidence regarding the lack of palliative care among COPD patients. A large retrospective study found that COPD patients received less palliative care, and the timing of palliative care among COPD patients is much later in the course of the disease compared to lung cancer [17]. They also found that more COPD patients end up dying in an institution or a nursing home. Difficulties in predicting disease trajectory among COPD patients were among the most common reasons for less palliative care involvement among COPD patients. They concluded that palliative care if used among COPD patients, is most likely to be used during the terminal stage before death. The reason could be the delay in doctor-patient communication regarding end-of-life care. 

The healthcare approach among COPD patients is often initiated to treat acute exacerbations rather than being initiated proactively based on the previously developed plan for managing the disease [17]. In the year 2018, Halpin emphasized how palliative care is still underutilized among COPD patients. They found that one in five patients with COPD receives palliative care, and just under half of them received it within the last six months and only one-third in the previous month of their life [21]. This raised a question regarding insufficient documentation of palliative care being received by these patients implicated in other studies. Nevertheless, the magnitude of under documentation is less significant than the underutilization of palliative care among COPD patients [21].

Common barriers related to palliative care involvement in COPD patients

Underrecognition of palliative care needs of the COPD patient by the physicians is one of the most common reasons for the unmet needs of COPD patients. For example, Mefferts et al., in their study, found that only 9.1% of the COPD patients admitted to the hospital were identified by the discharging physician as having palliative care needs. Moreover, around 50% of these patients had an underlying diagnosis of cancer [22]. This suggests that most of these patients had palliative care, not due to COPD but due to cancer [22]. Thus, palliative care, commonly misconstrued as end-of-life care, not only by the patients and their caregivers but also by the health care professionals, is one of the primary reasons for the lack of these services among COPD patients [23]. 

A nationwide analysis of the use of palliative care predicted that there are significant barriers to access palliative care, which included race, socioeconomic status, hospital size, and region of the country. They found that the black population, lower-income group, and smaller hospitals were less likely to receive palliative care consultation during their index hospitalization compared to Caucasians, higher-income group, and large hospitals, respectively. Rural hospitals have poor access compared to urban, and teaching hospitals have better access to palliative care than non-teaching hospitals. Even though palliative care involvement is endorsed for these patients by most respiratory societies worldwide, the vast majority of patients do not receive palliative care services as an inpatient in times of crisis. The increased referral to palliative care seen in larger teaching hospitals likely reflects subspecialty palliative care services in these hospitals compared with smaller rural hospitals. Frequently, rural hospital settings have under-resourced palliative care services to support COPD patients due to the frequent longevity of their needs, unlike cancer patients that tend to have shorter overall survival [23]. Complete dependence on a dedicated palliative care team may not be feasible across all the spectrums of healthcare. Therefore all physicians involved in the care of patients with COPD should recognize and address the unmet needs of these patients.

Palliative care reducing readmissions

Even though studies directly evaluating the relationship between palliative care involvement and COPD readmissions are scarce, multiple studies have looked at the impact of inpatient palliative care on the readmissions in mixed population studies. Selected relevant studies are briefly compared in Table 2. Gade et al. were among the first to conduct a multicenter randomized controlled trial with a sample size of 517 patients. The intervention group received interdisciplinary palliative care services (IPCS) consultation for palliative care. In contrast, the control group received usual care [6]. The results showed improved hospital admissions and readmissions, including the patient satisfaction score and increased utilization of hospice services. There was also a significant reduction in hospital costs. Nelson et al., in 2011, in a single-center small sample size study, found that involving the multi-disciplinary end-of-life care team compared to single palliative consult yielded better readmission rates and cost outcomes. However, they did not account for the timing of palliative care intervention and the comorbid illnesses [17]. This was followed by other studies, including the nationwide inpatient sample analysis by O'Connor et al. in 2015, where 4.1% (1430) of 34541 patients received inpatient palliative care consultations, and the propensity-matched scoring indicated that those with IPCs had lower 30-day readmissions (AOR: 0.66) compared to patients who did not receive it. An interesting finding of this study was that among the patients who had IPC, the ones who had goals of care discussion had a better odds ratio of 0.33 for readmission than those who only had palliative care for symptom control. This highlights the importance of GOC establishment to reduce readmissions [24,25]. Similar improvement in readmission rates was seen in further studies conducted by Bharadwaj (2016) and Wiskar (2017) in their multicenter retrospective studies. Wiskar et al. looked at the direct association between IPC and heart failure readmissions, which showed improved readmissions and costs associated with palliative care. Like COPD, heart failure is also a nonmalignant extended life-limiting disease with a high symptom burden and reduced quality of life [26,27].

**Table 2:** Studies showing the impact of inpatient palliative care on the readmissions

Author	Year	Journal	Study design	Sample size	Parameters measured	Results/comments
Gade [6]	2008	J palliative med	Multicenter RCT	517; 66 (COPD)	IPC targeted all patients	Improved readmission rate and lower admission costs
Nelson [17]	2011	Permanente Journal	Single-center retrospective	200; 100 (seen by only the Inpatient palliative care nurse) 100(multi-disciplinary palliative care team approach)	6-month hospital readmissions	readmissions to the hospital per patient per six months after consultation with multi-disciplinary team decreased from 1.15 to 0.7 admissions per patient
O'Connor [24]	2015	J palliative med	NRD	34541 ; 1430(PCC)	Propensity score matching	Lower 30-day readmission AOR (0.66) goals of care discussion yielded better OR (0.33) compared to symptom management (1.02)
Bhardwaj [27]	2016	J palliative med	Multicenter retrospective study		Overall outcomes with involvement of IPC	Reduced 30, 60, 90-day readmissions, lesser ICU LOS, lesser costs, higher hospice referral
Wiskar [26]	2017	J internal med	Nationwide retrospective study	102,746	HF readmissions, propensity score matching	Improved readmissions and costs associated with palliative care consults
Chuang [28]	2017	J palliative med	A single-center retrospective study	8215 (712)	CHF readmissions Propensity matching	No reduction in risk of 30-day readmissions
Zalenski [29]	2017	J pain and symptom management	Prospective Quality Improvement	405	Propensity score matching 161(PCC)VS 244(UC)	No change in readmission rates more patients with MOLST charting, better LOS and cost outcomes
Barkley [30]	2018	J palliative med	Single health system with eight hospitals retrospective observational study	49506; 43463(non-PCC) 6043(PCC)	Impact of timing on palliative care consults	Earlier PCC reduces readmissions and mortality

In addition, COPD and congestive heart failure co-exist in the same patients frequently. Hence, the involvement of palliative care and its effect on the outcomes of heart failure readmissions can be extrapolated in terms of COPD readmissions. However, Chuang et al., in their single-center retrospective study, could not replicate the similar outcomes as the previous studies, when the involvement of palliative care did not show any reduction in readmission rates in their study [28].

A prospective quality improvement project by Zalenski et al. did a similar study as by Chuang et al., among ICU patients [28,29]. Unfortunately, in the study, palliative care involvement did not reduce readmission rates, even though there was a significant improvement in cost outcomes, reduced length of stay, and improved advance care directives [29]. These studies with less encouraging results raised a question on the timing of involvement of palliative care services during their hospital length of stay, which was tested in a single system multihospital retrospective analysis by Barkeley et al. in 2018. They studied 6043 patients with palliative care and compared them to 43463 patients with non-palliative care and found that earlier palliative care involvement during the hospital stay led to reduced readmission rates and significant improvement in overall mortality rates [30].

Tangeman et al. found that patients with IPC consults had lower readmissions rates when they were discharged with hospice care. However, the rates were significantly higher among palliative care patients discharged to non-hospice services [31].

Approach towards COPD readmissions

The care of COPD should be comprehensive and multi-disciplinary with the involvement of palliative care early on. Comprehensive support for emotional, spiritual, and physical needs is more than just terminal end-of-life care. It is focused on patients and the caregivers to educate them about the progression of the disease and be prepared for things to expect in the future. Determination of advance directives and easing the suffering through establishing good support by involving palliative care is an important initiative [32]. Open discussion about death is vital to mitigate patients' fears and to allow them to make informed decisions regarding end-of-life care or in the event of a change in a medical condition that may prompt for hospital visit [33].

The COPD readmission rates can be influenced by the involvement of palliative care and hospice services. Hospitals that have their patients heavily relying on palliative and hospice teams may have different COPD readmission rates than hospitals that do not have a dedicated palliative care team or a heavy institution of hospice services [14]. Cherlin et al. enumerated three crucial strategies based on their survey of the State Action on Avoidable Readmissions (STAAR) initiative to facilitate hospice and palliative care involvement in reducing readmissions. This included designing and implementing tracking systems to identify at-risk patients for readmissions, providing education to the families and clinical staff, and establishing links to post-hospital settings [14]. Similarly, early involvement of palliative care in chronically ill patients has been shown to reduce readmissions and mortality, and this idea can be broadened to include COPD patients.

Early Palliative care evaluation for patients with higher readmission risk scores like LACE score>10 (L=length of stay, A=acuity, C=comorbidities, E=Emergency Department visits within the last six months) can be used as one of the tools in reducing COPD burden in hospital. Providing home palliative care services to patients who are being discharged from hospital with COPD might help cope patients and families with the idea of chronicity of the disease and how to handle it in the last days. Many studies are being done on pulmonary rehabilitation and its importance in reducing exacerbation rates in COPD [34]. Rehabilitation and palliative care have many similarities. Both of these specialties are affiliated with a multi-disciplinary approach. The common goal is to obtain an optimal functional status and quality of life. On the one hand, pulmonary rehabilitation focuses mainly on improving the patient's functional status, whereas palliative care focuses majorly on symptom relief and improving the quality of life. Both of these specialties incorporate family members in the care of patients. Patients can be referred by pulmonary rehab to palliative care if their symptoms reach beyond control or the disease continues to worsen [21]. With the help of advance care directives and the establishment of care goals, palliative care can empower the patients and their primary physicians to seek alternatives other than hospitalization for acute changes in medical conditions. This can help reduce the admission burden in prolonged serious illnesses like COPD.

## Conclusions

The existing data is limited to state that the institution of palliative care among COPD patients can reduce COPD-related readmissions. Nevertheless, when palliative care is involved in an early stage of the disease and with hospice enrollment, it can be associated with improved readmission rates. Therefore, future studies should aim at evaluating this relationship, specifically with the involvement of inpatient palliative services.
